# *In vivo* Evidence for Therapeutic Properties of Cannabidiol (CBD) for Alzheimer's Disease

**DOI:** 10.3389/fphar.2017.00020

**Published:** 2017-02-03

**Authors:** Georgia Watt, Tim Karl

**Affiliations:** ^1^Karl Group, Behavioural Neuroscience, Western Sydney UniversityCampbelltown, NSW, Australia; ^2^Neuroscience Research AustraliaRandwick, NSW, Australia

**Keywords:** Alzheimer's disease, cannabidiol, Δ^9^-tetrahydrocannabinol, transgenic mouse model, therapy

## Abstract

Alzheimer's disease (AD) is a debilitating neurodegenerative disease that is affecting an increasing number of people. It is characterized by the accumulation of amyloid-β and tau hyperphosphorylation as well as neuroinflammation and oxidative stress. Current AD treatments do not stop or reverse the disease progression, highlighting the need for new, more effective therapeutics. Cannabidiol (CBD) is a non-psychoactive phytocannabinoid that has demonstrated neuroprotective, anti-inflammatory and antioxidant properties *in vitro*. Thus, it is investigated as a potential multifunctional treatment option for AD. Here, we summarize the current status quo of *in vivo* effects of CBD in established pharmacological and transgenic animal models for AD. The studies demonstrate the ability of CBD to reduce reactive gliosis and the neuroinflammatory response as well as to promote neurogenesis. Importantly, CBD also reverses and prevents the development of cognitive deficits in AD rodent models. Interestingly, combination therapies of CBD and Δ^9^-tetrahydrocannabinol (THC), the main active ingredient of *cannabis sativa*, show that CBD can antagonize the psychoactive effects associated with THC and possibly mediate greater therapeutic benefits than either phytocannabinoid alone. The studies provide “proof of principle” that CBD and possibly CBD-THC combinations are valid candidates for novel AD therapies. Further investigations should address the long-term potential of CBD and evaluate mechanisms involved in the therapeutic effects described.

## The problem

Alzheimer's Disease (AD) is a debilitating neurodegenerative disease that is characterized by cognitive decline. It is the most common form of dementia, accounting for over 60% of cases and affecting over 33 million people worldwide (Wisniewski and Goni, 2014; Alzheimer's Association, 2015). Unfortunately, as a result of the aging population, this number is expected to reach 115 million by the year 2050 (Wisniewski and Goni, 2014). AD typically begins with mild deficits in short-term memory, learning, communication and spatial orientation. In the moderate stage of the disease, the deficits begin to affect everyday life including eating, dressing and emotional control (Alzheimer's Association, 2015). In the late stages of the disease there is global disruption of cognitive ability, with severe impairments in speech and facial recognition, all of which renders the patients in need of 24-h care. As the disease progresses, patients become increasingly susceptible to other illnesses as well (Alzheimer's Association, 2015).

## The origin

AD is classified into two types, late-onset sporadic AD (>95% of cases) or early-onset familial AD (<5% cases) (Gotz and Ittner, 2008). Although, sporadic AD is the most common form, it is much less understood than familial AD. Familial AD is also known as the genetic form, as it results from autosomal dominant mutations in the amyloid precursor protein (*APP*) gene or in the presenilin 1 and 2 (*PS1* and *PS2*) genes (Gotz and Ittner, 2008; Bettens et al., 2013). APP is the precursor molecule, which is cleaved into amyloid-β (Aβ) peptides, while *PS1* and *PS2* encode the γ-secretase and β-secretase complexes that mediate APP splicing (Bettens et al., 2013; Gotz and Ittner, 2008). After APP splicing Aβ can exist in two forms, Aβ_40_ and Aβ_42_. Aβ_42_ is thought to be the more toxic form of the protein as it aggregates more readily than Aβ_40_ (Chapman et al., 2001). The cause of sporadic AD is less clear and yet to be defined, however, recent research indicates that it may result from a complex interaction between several environmental factors and various susceptible genes. Numerous genes have been reported as susceptible genes for sporadic AD with the best-documented one being *APOE* (Kamboh, 2004).

Although familial and sporadic AD differ in their cause, the progression of the disease from this point onwards appears to be the same. Both forms of AD exhibit a neurodegenerative cascade that appears to be instigated by the accumulation of Aβ (forming senile plaques) and hyperphosphorylated tau [forming neurofibrillary tangles (NFTs)] (Chapman et al., 2001). The cascade induces neuroinflammation and oxidative stress, which creates a neurotoxic environment that potentiates neurodegeneration and eventually leads to cognitive decline (Hardy and Selkoe, 2002; Ahmed et al., 2015). Also, Aβ-induced neurodegeneration elevates glutamate levels in the cerebral spinal fluid of AD patients (Pomara et al., 1992) and cholinergic neurons are lost in brain areas relevant for memory processing (and accompanied by a decrease in acetylcholine) (Schliebs and Arendt, 2011).

## Current treatments

Despite the increase in our understanding of disease mechanism, the current approved AD treatments only provide limited therapeutic benefits. There are four approved drugs available, three are acetylcholinesterase inhibitors (rivistagmine, donepezil and galantamine) and one is a N-methyl-D-aspartate (NMDA) receptor antagonist (memantine) (Mangialasche et al., 2010). Unfortunately, all of them have been associated with adverse effects. Acetylcholinesterase inhibitors may cause nausea, vomiting, diarrhea and weight loss (Kaduszkiewicz et al., 2005), while memantine is known to cause hallucinations, dizziness and fatigue (Herrmann et al., 2011). Furthermore, none of these treatments prevent or reverse the progression of the disease but rather they treat the disease symptoms with limited efficacy (Salomone et al., 2012).

Current clinical trials to evaluate new AD treatments are targeting various aspects of AD pathology, with a strong focus on Aβ. Clinical trials have investigated both β- and γ-secretase inhibitors, which play a crucial role in the formation of pathological Aβ. Unfortunately, β-secretases are difficult to target and γ-secretases have a wide range of functions resulting in adverse side effects (e.g., impaired cognition and functionality, gastrointestinal toxicity and increased incidence of skin cancer) (Imbimbo and Giardina, 2011; Schenk et al., 2012). Active and passive immunotherapies to target senile plaques and NFTs have also been investigated. Aβ immunotherapies in mouse models demonstrated potential as they increased microglial phagocytosis of Aβ and reduced cognitive decline. However, in phase II and III clinical trials those therapies have demonstrated limited efficacy or resulted in severe adverse effects (e.g., meningoenchephalitis) (Mullane and Williams, 2013). A recent study investigating an antibody based immunotherapy for Aβ found promising results in phase I and phase II trials but this therapy is yet to undergo phase III clinical trials (Sevigny et al., 2016). Tau immunotherapies were effective in AD mouse models but have provided limited success in clinical trials (McGeer et al., 2006; Schenk et al., 2012; Mullane and Williams, 2013).

Epidemiological data have shown that non-steroidal anti-inflammatory drugs (NSAIDs) are associated with a reduced risk of AD (McGeer et al., 2006). Furthermore, animal studies indicated that NSAID treatment could attenuate AD pathogenesis, proposing that inhibiting neuroinflammation may slow the progression of AD (Maccioni et al., 2009). However, NSAIDs have also been associated with severe long-term adverse effects (e.g., gastrointestinal problems) and have only shown limited efficacy in reducing or preventing clinical symptoms (McGeer et al., 2006; Rojo et al., 2008).

It is unlikely that any drug acting on a single pathway or target will mitigate the complex pathoetiological cascade leading to AD. Therefore, a multifunctional drug approach targeting a number of AD pathologies simultaneously will provide better, wider-ranging benefits than current therapeutic approaches (Van der Schyf and Geldenhuys, 2011; Bedse et al., 2015). Importantly, the endocannabinoid system has recently gained attention in AD research as it is associated with regulating a variety of processes related to AD, including oxidative stress (Marsicano et al., 2002), glial cell activation (Germain et al., 2002) and clearance of macromolecules (Bilkei-Gorzo, 2012).

## Cannabidiol

The phytocannabinoid cannabidiol (CBD) is a prime candidate for this new treatment strategy. CBD has been found *in vitro* to be neuroprotective (Esposito et al., 2006b), to prevent hippocampal and cortical neurodegeneration (Hamelink et al., 2005), to have anti-inflammatory and antioxidant properties (Mukhopadhyay et al., 2011), reduce tau hyperphosphorylation (Esposito et al., 2006a) and to regulate microglial cell migration (Walter et al., 2003; Martín-Moreno et al., 2011). Furthermore, CBD was shown to protect against Aβ mediated neurotoxicity and microglial-activated neurotoxicity (Janefjord et al., 2014), to reduce Aβ production by inducing APP ubiquination (Scuderi et al., 2014) and to improve cell viability (Harvey et al., 2012) (summarized in Table 1). These properties suggest that CBD is perfectly placed to treat a number of pathologies typically found in AD. In the following, we will outline in brief the endocannabinoid system and the pharmacological profile of CBD before discussing recent advances in the evaluation of the therapeutic properties of CBD (and CBD-THC combinations) using *in vivo* AD rodent models.

**Table 1:** **Summary of the effects of CBD and CBD-THC combinations on AD models**.

**Effect of Cannabidiol on AD-like Pathology**		
**Model**	**Effect**	**References**
***IN VITRO*** **STUDIES USING CBD**		
PC12 Neuronal Cells	Protected against Aβ neurotoxicity and oxidative stress, increased cell survival and decreased ROS production and lipid peroxidation	Iuvone et al., 2004
	Inhibited tau hyperphosphorylation	Esposito et al., 2006a
	Prevented transcription of pro-inflammatory genes	Esposito et al., 2006b
Glutamate Neuronal Toxicity Model	Antioxidant properties	Hampson et al., 1998
Primary Rat Microglia	Increased microglial migration and prevented ATP-induced intracellular calcium increase	Martín-Moreno et al., 2011
PC12 and SH-SY5Y Cells	Improved cell viability after treatment with *tert*-butyl hydroperoxide treatment	Harvey et al., 2012
SH-SY5Y Cells	Protected against Aβ neurotoxicity and microglial-activated neurotoxicity	Janefjord et al., 2014
SH-SY5Y^APP+^ Cells	Induced APP ubiquination and subsequently Aβ production and increased cell survival by reducing apoptotic rate	Scuderi et al., 2014
***IN VIVO*** **STUDIES USING CBD**		
Mice inoculated with human Aβ_42_ peptide	Attenuated Aβ induced neuroinflammatory responses by decreasing expression of pro-inflammatory gene and mediators	Esposito et al., 2007
	Reduced reactive gliosis	Esposito et al., 2011
Mice intraventricularly injected with fibrillar Aβ	Decreased microglial activation and reversed a spatial reference memory deficit in the MWM	Martín-Moreno et al., 2011
*APPxPS1* transgenic mice (mixed background)	Reversed social and object recognition memory deficits in the CB task	Cheng et al., 2014a
	Prevented development of social recognition memory deficits. No effect on Aβ load but subtle effects on inflammatory markers, cholesterol and dietary phytosterol retention	Cheng et al., 2014b
***IN VIVO*** **STUDIES USING CBD-THC**		
Young *APPxPS1* transgenic mice (mixed background)	Improved memory deficits in the two-object recognition task and the active avoidance task. Decreased soluble Aβ_42_ levels and changed plaque composition and reduced astrogliosis, microgliosis and inflammatory related molecules	Aso et al., 2015
Aged *APPxPS1* transgenic mice (mixed background)	Restored cognition in the two object recognition task but had no effects on Aβ load or related glial reactivity	Aso et al., 2016
Transgenic tauopathy mouse model	Reduced Aβ and tau deposition in the hippocampus and cerebral cortex, increased autophagy, decreased gliosis, increased the ratio of reduced/oxidized glutathione and reduced levels of iNOS	Casarejos et al., 2013

### The endocannabinoid system and CBD pharmacology

The endocanabinoid system (eCBS) consists of endocannabinoids [e.g., anandamide and 2-arachiodonoylglycerol (2-AG)], enzymes required for their synthesis and degradation [fatty acid amide hydrolase (FAAH), monoglyceride lipase (MAGL), and diacylglycerol lipase (DAGL)], and cannabinoid receptors [the best described being cannabinoid receptors 1 and 2 (CB_1_ and CB_2_)], (Di Marzo et al., 2015). Post mortem analyses have found that several of these components are altered in both composition and signaling in AD postmortem brain tissue (Aso and Ferrer, 2015).

CBD has a complex interaction with the eCBS. It has demonstrated low displacement at the CB_1_ and CB_2_ receptors compared to other cannabinoids such as Δ^9^-tetrahydrocannabinol (THC) (Thomas et al., 1998). CBD has also been shown to have low affinity for both cannabinoid receptors (Petitet et al., 1998) and has antagonistic properties against the synthetic cannabinoid, CP 55 940, which is a potent agonist at both CB_1_ and CB_2_ receptors. Interestingly, CBD antagonizes CP 55, 940 at a much lower concentration than it binds to the cannabinoid receptors, suggesting it may act at a prejunctional site which is not the cannabinoid receptors (Pertwee et al., 2002). CBD acts as an inverse agonist at the CB_2_ receptors, which may explain some of its anti-inflammatory properties as inverse agonists at CB_2_ receptors are able to inhibit the migration of immune cells (Lunn et al., 2006). CBD has also been found to act as an antagonist at the cannabinoid G-protein receptors (GPR) GPR55 and GPR18 (Ryberg et al., 2007; McHugh et al., 2010), as well as activate the putative abnormal CBD receptor (Pertwee, 2008) and the vanilloid receptor 1 (Bisogno et al., 2001). Finally, CBD interacts with various neurotransmitter systems including glutamate receptors [i.e., NMDA receptors, 2-amino-3-(4-butyl-3-hydroxyisoxazol-5-yl)propionic acid (AMPA) receptors and kainite receptors] and the serotonergic receptor, 5-HT_1A_ (Russo et al., 2005). The wide range of targets of CBD emphasizes its potential as a multimodal drug for AD treatment.

### CBD effects in pharmacological rodent models of AD

The *in vivo* therapeutic potential of CBD in AD has not been widely documented, however, there are a number of studies that have reported the effect of CBD in pharmacological models of AD (e.g., inoculation with fibrillar Aβ). These studies have described anti-inflammatory and neuroprotective effects of CBD. The *in vivo* anti-inflammatory effects of CBD were confirmed in a mouse model of AD where the mice were intrahippocampally injected with human Aβ_42_ and then treated daily with intraperitoneal (i.p.) injections of CBD (2.5 or 10 mg/kg) for 7 days (Esposito et al., 2007). The results from this study demonstrated that CBD was able to dose-dependently inhibit glial fibrillary acidic protein (GFAP) mRNA and protein expression. GFAP is the best known marker of activated astrocytes and thought to be one of the main features of reactive gliosis (Esposito et al., 2007). Therefore, these results imply that CBD is able to reduce Aβ-induced reactive gliosis. In addition, CBD reduced both iNOS and interleukin-1β (IL-1β) protein expression and the related NO and IL-1β release (Esposito et al., 2007). NO and IL-1β are a few of the many active substances released by Aβ-stimulated microglia and therefore have been identified as potential modulators of neuronal damage. NO is a free radical and important in neuroinflammatory and neurodegenerative conditions, which include accelerating protein nitration and increasing tau hyperphosphorylation (Esposito et al., 2007). IL-1β is involved in the cytokine cycle responsible for neurodegeneration, the synthesis and processing of APP, the activation of astrocytes and the overexpression of iNOS and overproduction of NO (Esposito et al., 2007). Data from *in vitro* studies suggest that CBD may be able to reduce iNOS protein expression and NO release as a result of its ability to rescue the Wnt/β-catenin pathway, which plays a role in tau hyperphosphorylation (Esposito et al., 2006a). Finally, the ability of CBD to attenuate reactive gliosis may result from CBD's ability to act as an inverse agonist at the cannabinoid receptor 2 (CB_2_), which is thought to be involved in reactive gliosis (Walter and Stella, 2004; Thomas et al., 2007).

The anti-inflammatory and neuroprotective effects of CBD were further investigated in a rat model of AD-related neuroinflammation. This study evaluated the involvement of the peroxisome proliferator activated receptor (PPAR) receptors in the therapeutic effects of CBD, as PPAR-γ receptors are increased in AD patients (Esposito et al., 2011). Adult, male rats were inoculated with human Aβ_42_ in the hippocampus and then treated with CBD (10 mg/kg) either in the presence or in the absence of a PPAR-γ or PPAR-α receptor antagonist for 15 days. CBD was able to dose-dependently decrease Aβ-induced expression of iNOS, GFAP, S100 calcium binding protein B (S100B) and p50 and p56 antibodies in rat astrocytes (Esposito et al., 2011). iNOS and GFAP, as mentioned previously, are key elements in reactive gliosis and therefore their reduction demonstrates CBD's anti-inflammatory properties. CBD's ability to reduce reactive gliosis is further emphasized by the inhibition of S100B. S100B is an astroglial-derived neurotrophin that plays a crucial role in the pro-inflammatory cytokine cycle and the promotion of APP to cleave Aβ_42._ It is also involved in the disruption of the Wnt/β-catenin pathway and therefore inhibits tau hyperphosphorylation (Esposito et al., 2011). Furthermore, the reduction of p50 and p56 expression indicates CBD's ability to inhibit NF-κB and therefore emphasizes the responsibility of both PPAR-γ and NF-κB in CBD's anti-inflammatory properties (Esposito et al., 2011). The therapeutic benefit of CBD was blocked when co-administered with the PPAR-γ antagonist (but not the PPAR-α antagonist) (Esposito et al., 2011), suggesting that CBD-induced anti-inflammatory properties are mediated (at least partially) through the PPAR-γ receptor (Esposito et al., 2011). Finally, the study found that CBD was able to restore CA1 pyramidal neurons to a similar integrity to that of the control rats. CBD also down-regulated gliosis and repaired neurogenesis in the dentate gyrus (Esposito et al., 2011).

One study to date has investigated the effects of CBD on cognition in a pharmacological model of AD. Three-month old mice were intraventricularly injected with 2.5 μg of fibrillar Aβ. They were then treated with 20 mg/kg CBD using daily i.p. injections for 1 week and then 3 times/week for the following 2 weeks. The spatial learning of the mice was then assessed in the Morris Water Maze (Martín-Moreno et al., 2011). CBD treatment was able to reverse the cognitive deficits of Aβ-treated mice. Interestingly, selective CB_2_ agonists did not prevent the cognitive deficit, indicating that CBD exerts this therapeutic effect via other mechanisms (Martín-Moreno et al., 2011). CBD treatment also prevented Aβ-induced IL-6 gene expression suggesting that the behavioral benefits documented may be mediated by glial activation modulation. However, CBD did not influence TNF-α gene expression. *In vitro* results from this study supported this finding as CBD treatment prevented the ATP-induced intracellular calcium increase and promoted microglial activation in cultured microglia (Martín-Moreno et al., 2011).

### CBD effects in transgenic mouse models of AD

Although pharmacological models of AD are useful in producing AD-like symptoms, it is necessary to investigate the effects of CBD in transgenic mouse models as they result from gene mutations, which are seen in familial AD (e.g., *APP, PS1*, and *PS2* gene mutations). Furthermore, based on the pharmacological protocols used, some effects of CBD could be related to a direct effect of the phytocannabinoid on exogenous Aβ administration rather than the long-term effects of the accumulated Aβ. Initially, two studies were conducted in our laboratories to elucidate the remedial and preventative potential of chronic CBD treatment in AD transgenic mice. To assess the remedial effects of CBD, adult male *APPxPS1* mice were treated for 3 weeks with CBD (20 mg/kg CBD, daily i.p. injections) post onset of cognitive deficits and AD pathology (Cheng et al., 2014a). CBD treatment was able to reverse cognitive deficits in object recognition memory and social recognition memory without influencing anxiety parameters (Cheng et al., 2014a).

In the preventative treatment study, male *APPxPS1* mice at the age of 2.5 months were treated for 8 months with either 20 mg/kg CBD or vehicle pellets using a daily voluntary oral administration protocol (Cheng et al., 2014b). This assessed the long-term effect of CBD prior to “AD onset.” Long-term CBD treatment was able to prevent the development of social recognition memory deficits without affecting anxiety domains in AD transgenic mice (Cheng et al., 2014b). These beneficial effects were not associated with a reduction in Aβ load or oxidative damage. There was also no difference in hippocampal or cortical soluble and insoluble levels of Aβ_40_ and Aβ_42_ in the AD transgenic mice regardless of treatment. Furthermore, cortical lipid oxidation levels were not altered by CBD treatment. However, the study did report a complex interaction between CBD treatment, AD genotype and cholesterol and phytosterol levels, suggesting they may be involved in the mechanisms behind the beneficial effects of CBD. There was also a subtle impact of CBD on inflammatory markers of the brain (Cheng et al., 2014b). Further research will be necessary to elucidate the potential mechanisms further, thereby also considering other treatment designs (i.e., different ages at treatment onset and CBD doses).

Recent research has indicated that a combination of CBD and Δ^9^-tetrahydrocannabinol (THC) can avoid the detrimental effects caused by THC-induced activation of the CB_1_ receptors (e.g., psychoactivity), and actually provide greater therapeutic benefits than either phytocannabinoid alone. Importantly, there is controversy about what the ratios of CBD:THC should be used in order to antagonize detrimental THC effects. It has been reported that a >10-fold higher dose of CBD was necessary to prevent the unwanted side effects of THC. Other research suggests that CBD may even modestly potentiate THC's psychoactive effects (Fadda et al., 2004; Klein et al., 2011). Nevertheless, Sativex (GW pharmaceuticals, Salisbury, United Kingdom), a combination therapy using a 1:1 ratio of CBD and THC is approved as an anti-inflammatory drug treatment against spasms in multiple sclerosis and does not appear to be associated with any adverse THC effects, suggesting that CBD effectively blocks those at the ratio chosen (Collin et al., 2010; Novotna et al., 2011).

Three studies to date have evaluated the efficacy of a combination of CBD and THC on AD-related processes *in vivo*. The first study conducted by Casarejos et al. (2013) investigated the effects of Sativex in a mouse model of tauopathy. This mouse model was foremost a model of frontotemporal dementia, parkinsonism and lower motor neuron disease. The study found that Sativex decreased gliosis, increased the ratio of reduced/oxidized glutathione and reduced the levels of iNOS (Casarejos et al., 2013), thereby showing neuroprotective and anti-oxidant properties. Importantly, Sativex reduced Aβ and tau deposition in the hippocampus and cerebral cortex as well as increasing autophagy (Casarejos et al., 2013), thus implying, that although the mouse model is not directly related to AD, the therapeutic benefits are.

The second study conducted by Aso et al. (2015) compared the effect of CBD, THC and a CBD-THC combination in the *APPxPS1* mouse model, in the early symptomatic phase (~6 months). This study found that all treatments improved memory deficits in the two-object recognition task but only the CBD-THC combination prevented the learning deficit seen in the active avoidance task. CBD-THC combination also decreased soluble Aβ_42_ levels and changed plaque composition while CBD and THC individually did not (Aso et al., 2015). Finally, reduced astrogliosis, microgliosis and inflammatory related molecules were more pronounced after treatment with the CBD-THC combination than either phytocannabinoid individually (Aso et al., 2015). This suggests that when CBD and THC are combined there may be either a summative effect or an interaction effect between the compounds, which potentiates their therapeutic-like effects (Aso et al., 2015). In this context, it should be mentioned, that although all treatments had cognition-improving characteristics in the object recognition task, THC alone had a detrimental effect on cognition in control mice, highlighting the need to be cautious when considering THC as a therapeutic. However, control mice treated with CBD-THC combination did not show any cognitive deficits suggesting that CBD may be able to antagonize the detrimental effects of THC (Aso et al., 2015).

In a very recent follow-up study, Aso et al. also investigated the effect of CBD-THC combination treatment on memory and brain pathology in aged male *APPxPS1* mice and littermate controls (12 months) as well as non-aged controls, 3 months old control mice (Aso et al., 2016). Compared to the non-aged controls, vehicle-treated aged mice demonstrated impaired cognition in the two-object recognition task. Interestingly, CBD-THC combination restored the memory deficit of *APPxPS1* but not WT control mice (Aso et al., 2016). In comparison to their previous study testing younger *APPxPS1* mice (Aso et al., 2015), CBD-THC combination did not influence the Aβ load or the related glial reactivity in aged AD transgenic mice (Aso et al., 2016)., However, the combination treatment normalized synaptosome associated protein 25, glutamate receptors 2 and 3 and γ-aminobutyric acid receptor A subunit α1 expression, implying that CBD-THC may exert its beneficial effects on cognition via these mechanisms.

## Conclusions

AD is a debilitating neurodegenerative disease that is becoming increasingly common in today's society. Unfortunately, there is still no effective treatment that stops or reverses the disease progression. The studies reviewed in this mini review provide “proof of principle” for the therapeutic benefits CBD and possibly CBD-THC combinations pose for AD therapy (summarized in Table 1). However, further dose-dependent investigations into transgenic mouse models of AD are necessary to understand the full potential and the long-term effects of CBD. Importantly, many of the discussed studies were conducted in mice aged between 3 and 6 months, which is quite young considering AD diagnosis is usually relatively late in the disease progression. Furthermore, it is necessary to investigate the effects of CBD in tauopathy mouse models specific to AD and in female mouse models as all studies reviewed were conducted in male mice only. Nevertheless, the studies discussed here provide promising preliminary data and the translation of this preclinical work into the clinical setting could be realized relatively quickly: CBD is readily available, appears to only have limited side effects (Bergamaschi et al., 2011) and is safe for human use (Leweke et al., 2012).

## Author contributions

TK and GW were both involved in the conceptualization, reference search, and writing of this mini review.

### Conflict of interest statement

The authors declare that the research was conducted in the absence of any commercial or financial relationships that could be construed as a potential conflict of interest.
